# Binding Pocket Optimization by Computational Protein Design

**DOI:** 10.1371/journal.pone.0052505

**Published:** 2012-12-27

**Authors:** Christoph Malisi, Marcel Schumann, Nora C. Toussaint, Jorge Kageyama, Oliver Kohlbacher, Birte Höcker

**Affiliations:** 1 Max Planck Institute for Developmental Biology, Tübingen, Germany; 2 Center for Bioinformatics, Quantitative Biology Center, and Department of Computer Science, University of Tübingen, Tübingen, Germany; Weizmann Institute of Science, Israel

## Abstract

Engineering specific interactions between proteins and small molecules is extremely useful for biological studies, as these interactions are essential for molecular recognition. Furthermore, many biotechnological applications are made possible by such an engineering approach, ranging from biosensors to the design of custom enzyme catalysts. Here, we present a novel method for the computational design of protein-small ligand binding named PocketOptimizer. The program can be used to modify protein binding pocket residues to improve or establish binding of a small molecule. It is a modular pipeline based on a number of customizable molecular modeling tools to predict mutations that alter the affinity of a target protein to its ligand. At its heart it uses a receptor-ligand scoring function to estimate the binding free energy between protein and ligand. We compiled a benchmark set that we used to systematically assess the performance of our method. It consists of proteins for which mutational variants with different binding affinities for their ligands and experimentally determined structures exist. Within this test set PocketOptimizer correctly predicts the mutant with the higher affinity in about 69% of the cases. A detailed analysis of the results reveals that the strengths of PocketOptimizer lie in the correct introduction of stabilizing hydrogen bonds to the ligand, as well as in the improved geometric complemetarity between ligand and binding pocket. Apart from the novel method for binding pocket design we also introduce a much needed benchmark data set for the comparison of affinities of mutant binding pockets, and that we use to asses programs for *in silico* design of ligand binding.

## Introduction

Computational protein design has advanced rapidly in recent years. A particularly exciting and dynamic area is the design of interactions between proteins and small molecule ligands. This includes the design of receptors that bind ligands of choice, which for example can be used as biosensors [1], as well as the design of enzymes that do not only bind a substrate, but also contain the catalytic machinery to process it [2]–[3]. In all these designs, an existing protein is used as a scaffold, and its binding pocket is altered or a new one is introduced that should interact with the target ligand.

With this approach, enzymes have been designed that catalyze chemical reactions for which no natural catalysts exist, such as a kemp eliminase [4]–[5], a diels-alderase [6], and a retro-aldolase [7]. It has also been used to design a metalloenzyme by repurposing parts of the already existing catalytic machinery in the scaffold protein, namely the reactivity of a zinc metal center to hydrolyze organophosphates [8]. Furthermore, similar methods have been applied to change substrate specificities as well as affinities. For example human guanine deaminase was changed to bind ammelide through the remodeling of a loop that now provides a key interaction to the new target substrate [9], the substrate specificity of gramicidin S synthetase was changed from phenylalanine to leucine [10], and mutations in dihydrofolate reductase from *Staphylococcus aureus* were predicted that decrease binding to an inhibitor molecule while stabilizing native protein function [11].

While these are impressive results, there is still much room for improvement in the computational methods. Specifically, it seems to be difficult to accurately design a protein for high affinity binding to a ligand or transition state [12]. The majority of the enzyme designs mentioned have low affinities for their substrates when compared to naturally occurring enzymes [13]–[14]. In a rare report of a failed attempt, the unsuccessful design of a high-affinity ligand binding site for a D-Ala- D-Ala dipeptide into an endo-1,4-xylanase scaffold was discussed. Designs by the employed design software Rosetta did not show the predicted high affinity in the experimental tests underscoring the challenge of protein-ligand interface design [15]. In this respect long-range electrostatics and dynamics, accurate modeling of solvation and electrostatics at the interface, as well as the inclusion of explicit water molecules have been named as most problematic areas [13]–[16]. In order to improve protein-ligand interface design and to overcome current limitations it will be necessary to test design protocols more systematically.

In this respect, we noticed that in computational design studies there is a lack of more general benchmark sets. Related molecular modeling techniques are regularly assessed using test sets. For example protein-ligand docking algorithms have been compared in detail [17]–[18] [19]–[20]. Also the CASP and CAPRI experiments allow unbiased testing of protein structure prediction and protein-protein docking methods [21]. In contrast only a few computational design studies tested their employed methodology. One example is the redesign of the binding pocket of ribose binding protein for its native ligand using molecular mechanics methods. Among the resulting binding pocket sequences, the wild type sequence was ranked second best, while the first and third ranks had only a single mutation and bound ribose with tenfold decreased affinity [22]. Also the aforementioned algorithm to introduce one key interaction to a ligand using loop modeling techniques was tested on eight proteins. For six of them the method produced a loop of the same length and similar configuration as in the crystal structures [9]. Both benchmark tests are very specific, they cannot be used to generally and systematically assess a method’s proficiency in designing binding to a small molecule. Also the broader benchmark set that was used to assess the ability of the enzyme design methods RosettaMatch and ScaffoldSelection to identify suitable scaffold proteins that can host a desired catalytic machinery [23]–[24] are not suited for this purpose. Such a test set, however, would be very helpful for assessing the potential and the shortcomings of available methods.

In this study, we present PocketOptimizer, a computational pipeline that can be used to predict mutations in the binding pocket of proteins, which increase the affinity of the protein to a given small molecule ligand. It can be used for the analysis of few mutations as well as for the design of an entire binding pocket. It uses several molecular modeling modules. Side chain flexibility is sampled by a conformer library, which we compiled following Boas and Harbury [22]. The use of conformer libraries has been reported to be advantageous, especially in the context of binding-site geometries [25] [26]–[27]. A receptor-ligand scoring function is used to calculate protein ligand binding strength. The modular architecture of PocketOptimizer allows easy and systematic comparison of methods that perform the same task. As the first test we utilize this to examine two scoring functions in this study, the scoring function provided by CADDSuite [28] and Autodock Vina [29]. In order to assess the performance of PocketOptimizer and other methods that address the same task, we compiled a benchmark set. It consists of mutational variants of proteins and their small ligands with available experimental structural and binding affinity data. We also used this benchmark to test the enzyme design application included in the Rosetta molecular modeling software. Rosetta was used for the majority of the design studies mentioned earlier, and it is the most successful freely available protein design software to date [30]. We find that both methods perform similarly. In our benchmark PocketOptimizer succeeds slightly better in predicting the correct affinity-enhancing mutations. We discuss the strengths and weaknesses of our method and describe to which protein design problems it can be applied with good chances of success. The findings emphasize the merit of a systematic approach to evaluate computational protein design methodologies, to identify their strengths, and to pinpoint possibilities for improvement. And our modular program PocketOptimizer provides a suitable framework to test and implement these approaches.

## Results and Discussion

### Computational Receptor Design Pipeline PocketOptimizer

**Figure 1:**
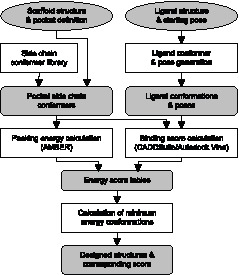
Workflow of PocketOptimizer. The input specific for a design is depicted in circles, parts of the pipeline are shown in pointed rectangles, and output components in rounded rectangles. The output is stored in standard file formats (SDF and PDB for structural data, csv for energy tables). This allows the easy replacement of a component with another that solves the same task (e.g. replacing the binding score function).

We developed PocketOptimizer for the design of protein-ligand interactions. In combination with a program such as ScaffoldSelection [24] it can also be used for enzyme design. PocketOptimizer is a combination of customizable molecular modeling components. Amino acid flexibility is modeled by a side chain conformer library, ligand flexibility is addressed by systematically sampling poses of the ligand in the binding pocket. The score that is optimized is a combination of protein packing energy calculated with the AMBER force field [31], and protein-ligand binding energy calculated using a scoring function. To identify the most promising design, the global minimum energy conformation of a protein pocket with the ligand based on the combined energy score is calculated [32]–[33]. Intermediate results like conformers or score tables are stored in standard file formats, making it easy to compare different approaches for a given subtask. Notably, we used two receptor-ligand scoring functions in this study, the scoring function included in CADDSuite [28] and Autodock Vina [29]. Figure 1 depicts the workflow of the PocketOptimizer pipeline.

The program PocketOptimizer is designed as a modular pipeline that allows exchange of program parts, e.g. the use of different available docking functions or force-fields. In contrast to other existing design programs this pipeline aims to provide a platform for the incorporation and testing of available modules so that the contribution of individual parts can be distinguished. In its current implementation of PocketOptimizer we chose to use a conformer library over rotamers. The program is geared towards the design of protein-ligand interaction, however it can also be used for prediction of protein packing only. Currently not incorporated are backbone flexibility and negative design capabilities.

PocketOptimizer source code and documentation can be obtained from the authors or from www.eb.mpg.de/research-groups/birte-hoecker/algorithms-and-software.html.

### Benchmark Set

**Figure 2:**
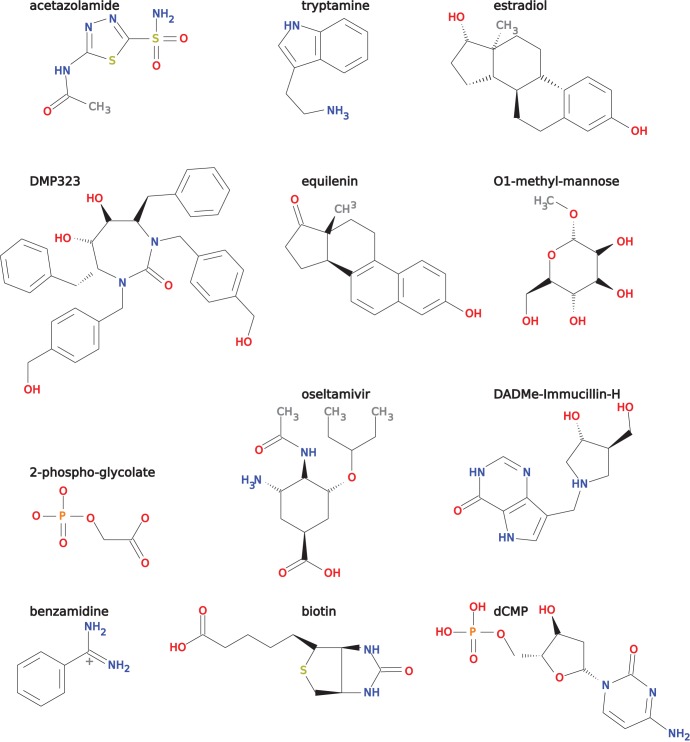
Two-dimensional structures of benchmark set ligands. The ligands of the test cases of our benchmark sets. See Table 1 for which ligand belongs to which test case.

We compiled a set of twelve proteins with structural and experimental affinity data for the assessment of computational design methods for protein-ligand binding. For this, we systematically searched the PDBbind database [34], which lists high quality crystal structures of protein-ligand complexes together with experimentally determined binding data. Each protein in our set has at least two mutational variants (usually the wild type and one or more mutants) accompanied by an affinity measure (the inhibitory constant 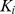 or dissociation constant 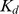) for the same ligand. The positions of amino acids that differ between the variants are always located in the binding pocket or active site. For each protein, there is at least one crystal structure of a variant with the ligand, for ten of the twelve there are two or more crystal structures that allow us to compare a design model of a variant with the respective crystal structure. The proteins and ligands in our benchmark set are very diverse. All ligands are shown in Figure 2. Each protein in the set belongs to a different fold as defined by SCOP [35], underscoring their structural diversity. This diversity allows to test design methods on a wide range of problems and avoids bias. Table 1 lists the benchmark proteins and their associated data.

**Table 1:** Benchmark set.

			Mutants		
Protein	Ligand	Positions	AA	aff. [nM]	PDB
			F	5.8	1ydb
			W	8.6	–
			L	9.6	–
			R	86	1ydd
Carbonic anhydrase II	Acetazolamide	198	E	280	1yda
			D	53	2pql
D7r4 amine binding protein	Tryptamine	111	L	inf	–
			E	0.29	1gwr
			Q	3.53	
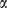 Estrogen receptor	Estradiol	353	A	60	–
			FIFI	0.4	1met
			VIVI	0.8	–
			VVVV	20	1mes
HIV-1 protease	DMP323	A:82, A:84, B:82, B:84	FVFV	800	1meu
			N	810	1ogx
Ketosteroid isomerase	Equilenin	240	D	45750	1oh0
			AG	2780	2jdn
Lectin II	O1-methyl-mannose	22, 24	SN	42900	2jdy
			H	2000	1egh
			N	5800	1s89
Methylglyoxal synthase	2-Phospho-gylcolate	98	Q	46000	1s8a
			H	0.32	2hu4
Neuroaminidase test 1	Oseltamivir	274	Y	84.8	3cl0
			N	0.32	2hu4
Neuroaminidase test 2	Oseltamivir	294	S	25.9	3cl2
			H	0.01	1rsz
			G	0.27	2a0w
			D	0.9	2a0y
Purine nucleoside phosphorylase	DADMe-Immucillin-H	257	F	0.95	2a0x
			N	0.0001	1swe
			E	0.0069	–
Streptavidin test 1	Biotin	s23	A	0.028	1n43
			S	0.0001	1swe
Streptavidin test 2	Biotin	27	A	0.01	1n9m
			C	490	1nja
			D	2800	1njc
Thymidylate synthase	dCMP	229	N	160000	1nje
			DG	12000	1ane
Trypsin	Benzamidine	189, 226	GD	15000000	1bra

Each row lists a test case. Columns **Protein** and **Ligand** contain the name of protein or ligand, **Positions** the indices of the mutable positions (for HIV protease along with the chain identifier, in the other cases the pocket is formed by one chain only), **Mutants** lists the variants: in subcolumn **AA** the amino acids at the mutable positions, in **aff.** the affinities of the variants, and in **PDB** the PDB identifier of the corresponding crystal structure, should one exist.

### Benchmark Results

**Figure 3:**
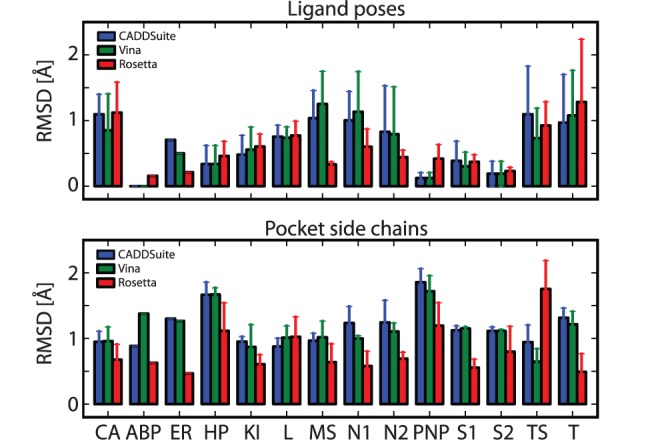
Differences of the ligand poses and pocket side chains in the benchmark designs compared to the crystal structures. The upper graph shows the average RMSDs and standard deviation between the ligand pose in the designs and in the crystal structures. The lower graph shows the average RMSD and standard deviation between the binding pocket side chain heavy atoms of designs and the corresponding crystal structure. The RMSDs are calculated after superimposing the structures using the backbone to make sure that the differences come from pocket/ligand pose differences only. RMSD from PocketOptimizer CADDSuite score designs are plotted in blue, from PocketOptimizer vina designs in green, and from Rosetta designs in red. Each point marks the average RMSD for all designs of a test case usign one score. The number of designs that contribute to a value depends on the number of mutations with a crystal structure, it is the square of this number (because each structure is used as a design scaffold for each mutation). Test cases are: *CA*: Carbonic anhydrase II, *ABP* D7r4 amine binding protein, *ER*: Estrogen receptor 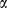, *HP*: HIV-1 protease, *KI*: Ketosteroid isomerase, *L*: Lectin, *MS*: Methylglyoxal synthase, *N1*: Neuroaminidase test 1, *N2*: Neuroaminidase test 2, *PNP*: Purine nucleoside phosphorylase, *S1*: Streptavidin test 1, *S2*: Streptavidin test 2, *TS*: Thymidylate synthase, *T*: Trypsin.

The optimization scheme of PocketOptimizer simultaneously chooses sequence and conformation. It can go over many alternatives. For the benchmark, however, it was necessary to restrict the sequence to the mutations for which experimental data was available. We tested the performance of PocketOptimizer on the benchmark set using Autodock Vina and CADDSuite receptor-ligand scores as well as Rosetta’s enzyme design application. Each method was used for the same set of design calculations. Each available crystal structure was used as a scaffold for the design of each mutational variant. We obtained a design for each mutation in each scaffold structure by forcing the methods to select a particular mutation in a separate run. This allowed us to compare the predicted binding and total energy scores as well as the designed conformations with the experimental data. Figure 3 shows the RMSD values between the designs and the respective crystal structures. This is a measure of how well the respective method models the conformation of the binding pocket residues and the ligand pose in the pocket. Rosetta performs better in modeling side chains in the binding pocket. The difference between the pocket RMSDs of Rosetta and each of the two PocketOptimizer variants is statistically significant with a p-value <0.01 according to a Mann-Whitney test. This might not come as a surprise considering that the Rosetta molecular modeling software is extensively used and optimized for protein packing tasks, especially protein structure prediction. PocketOptimizer on the other hand focuses on the identification of residues interacting favorably with the ligand. The observed differences in ligand pose RMSD are not statistically significant (Figure 3). To assess whether the methods can differentiate correctly between protein variants that have a large affinity difference, we looked at pairs that have an affinity difference of at least 50-fold. This cutoff translates to roughly 2.3 kcal/mole and was chosen to make sure that only pairs with clear, trustworthy affinity differences well outside experimental error are investigated. Table 2 lists the number of pairs in which the order of the mutants according to energy score is the same as the order according to affinity, meaning the design method would produce the correct ranking. Here, PocketOptimizer performs in the same range as Rosetta, with 69% correctly predicted pairs opposed to 64%. When comparing the two receptor-ligand score functions we used in our approach it seems that Autodock Vina has some advantage over the CADDSuite score. The total scores of the different methods are also listed. Based on these scores PocketOptimizer performs even better with 71% and 76% correctly predicted pairs. However, since we are looking at affinity prediction, the binding score appears to be more appropriate for the comparison.

**Table 2:** Order of designs by predicted binding score.

Test Cases	CADDSuite		Vina		Rosetta	
	Total	Binding	Total	Binding	Total	Binding
D7r4 amine binding protein	1/1	1/1	1/1	1/1	1/1	1/1
Estrogen receptor	1/1	1/1	1/1	1/1	1/1	1/1
HIV-1 protease	6/9	6/9	9/9	9/9	5/9	8/9
Ketosteroid isomerase	2/2	2/2	2/2	2/2	1/2	1/2
Neuroaminidase test 1	0/2	0/2	0/2	0/2	1/2	0/2
Neuroaminidase test 2	2/2	½	1/2	0/2	0/2	2/2
Purine nucleoside phosphorylase	6/8	6/8	7/8	6/8	4/8	2/8
Streptavidin test 1	4/4	4/4	4/4	4/4	3/4	3/4
Streptavidin test 2	2/2	2/2	2/2	2/2	2/2	1/2
Thymidylate synthase	1/6	0/6	1/6	0/6	6/6	3/6
Trypsin	1/2	½	1/2	1/2	0/2	1/2
**Mean**	**70.8%**	**65.6%**	**75.5%**	**68.8%**	**63.5%**	**63.5%**

**Numbers of correctly ranked design mutation pairs with large affinity difference.** All mutation pairs for which there is an affinity difference of at least 50-fold are investigated. All design pairs with these mutations (i.e. for each of these pairs there are as many design pairs as scaffold crystal structures) are checked, if the order of the mutations by total score or binding score is the same order as by affinity. A cell shows the number of correctly ordered design pairs, and the number of all design pairs. The mean for this part is calculated by scaling the percentage of a test case by the number of mutation pairs (i.e. NOT by design pairs, which would bias the value too much towards test cases with many crystal structures).

**Figure 4:**
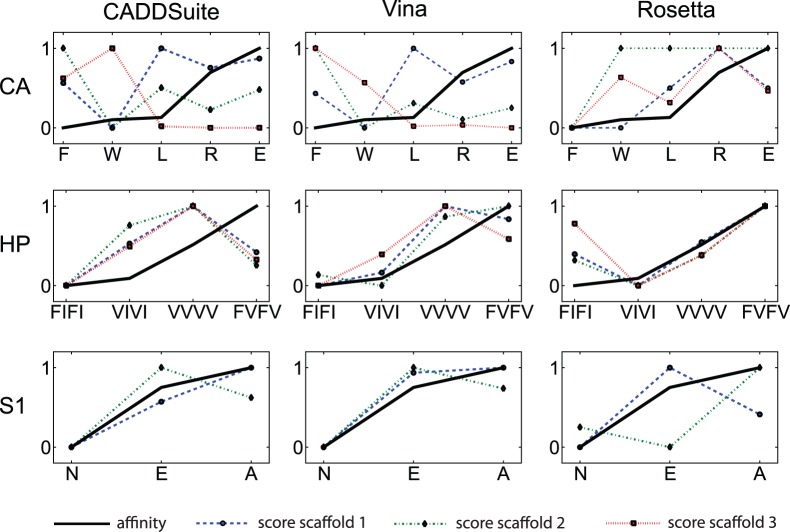
Comparison of the energy scores versus the affinities of the mutations show how well the programs reproduce the differences. For each test case with more than two mutations, we plotted the top binding scores of CADDSuite, Vina, and Rosetta designs for each mutation on each scaffold structure together with the logarithm of the affinity. Here we show plots for Carbonic anhydrase II, HIV-1 protease, and Streptavidin test 1. All other plots are shown in Information S1. Values are scaled to fit in the same range. Shown on the x-axis of a plot are the mutants in order of affinity to the ligand (the leftmost has the lowest affinity, compare Table 1 for the actual values). The y-axis measures predicted binding scores for the designs, and the log affinities, scaled between 0 and 1. Both are proportional to the binding free energy, and can therefore be compared when scaled to the same range. The lowest predicted binding score or log affinity is set to 0, the highest respective value to 1. Each plot contains a line for the affinity logarithm (solid, black no marker). This line represents the goal, if a method predicts binding well, the binding score lines should closely follow the log affinity line. The other markers and lines show the scaled predicted binding scores. One line represents the designs calculated for all available mutants, calculated by using one crystal structure as the scaffold. (Crystal structure 1: dashed, blue, circle markers; structure 2: red, dotted, square markers; structure 3: green, dash-dot pattern, diamond markers; structure 4: cyan, two dashes one dot pattern, star markers). We chose to use lines for representation, because this makes it easy to visually compare the shape of the black log affinity line to the lines representing the design binding scores. Each row has plots for one test case, in parentheses the order of scaffold structures is listed: *CA*: Carbonic anhydrase II (1ydb, 1yda, 1ydd), *HP*: HIV-1 protease (1met, 1meu, 1mes), *S1*: Streptavidin test 1 (1swe, 1n43).

We further examined how well the energy scores correlate with the affinities. For this we plotted the predicted energy of each design against the logarithmic affinities for all seven test cases with more than two mutations (Figure 4). The scores should correspond to the binding free energy, which in turn is proportional to the logarithm of the affinity of binding. Here, all mutants with experimental affinity values of a test case are included, regardless of the extent of the affinity difference. Overall we find that the energy values follow the affinity logarithm only in some cases.

### Discussion of Benchmark Results

When looking at a pair of protein variants, PocketOptimizer is able to correctly predict which variant has a better binding affinity if that difference is based on the introduction or abolition of a direct interaction of the mutable residue’s side chain with the ligand. This is especially noteworthy for pairs where one residue forms a hydrogen bond with the ligand, while the other does not. This was predicted correctly in seven of eight cases where the better binding variant forms an additional hydrogen bond. It also works well if the variable side chain of one mutation variant is bulkier than its counterpart in another variant, and therefore packs better against the ligand, i.e. forms more van der Waals (vdW) interactions with the ligand and shields it better from solvent, improving the solvation energy contribution. A potential downside of this effect of vdW contact improvement is that PocketOptimizer sometimes seems to prefer larger side chains even if they are detrimental to binding for other reasons. This tendency could lead to an overpacking of the designed pocket. When differences in binding have more complex causes, such as rearrangements in the pocket’s side chains that affect the ligand interaction indirectly by influencing other pocket side chains, the program generally fails to capture these differences.

Both scoring functions used within PocketOptimizer, from Autodock Vina and CADDSuite, produce results that are quite similar. The overpacking effect discussed before is less pronounced in Vina, which explains its slightly better performance in predicting which variant of a pair binds better (see Table 2). Generally, the order of the designs by energy scores calculated by our method does not depend on which variant’s crystal structure was used as the scaffold. Only in a few cases a significant difference can be observed, notably for carbonic anhydrase II and trypsin.

In some cases, the PocketOptimizer designs did not contain a conformational configuration that avoids vdW clashes in the binding pocket. In one test case, namely for neuroaminidase, the program was unable to identify any acceptable pocket conformation. One limitation of PocketOptimizer and a probable cause for such problems is the assumption of a fixed backbone in our designs. An adjustment of the backbone conformation might have helped to accommodate the tyrosine. It is also conceivable that our way of systematically sampling possible ligand poses could have failed to generate a pose that is sterically compatible in the neuroaminidase case.

Rosetta’s enzyme design application does not suffer from unresolvable vdW clashes. It includes minimization steps in its algorithm that can resolve potential clashes introduced by discrete conformational sampling. However, Rosetta apparently cannot convey its superiority in modeling the binding pocket side chains to the prediction of the correct binding score order. It is unable to predict the rearrangements of side chain conformations that lead to binding affinity changes in the more complicated test cases. The energy term for hydrogen bonds in Rosetta seems to have less influence on the output than in our program. This causes Rosetta to miss existing hydrogen bonds between ligand and side chains. The binding scores and their differences predicted for different mutants are more dependent on the scaffold structure used in Rosetta designs than it is in PocketOptimizer. This can be seen in Figure 4: the lines for designs of both PocketOptimizer variants, Vina and CADDSuite, are more similar to each other than the ones for Rosetta designs. This is rather surprising, as we anticipated that the limited backbone flexibility included in the Rosetta enzyme design protocol would lead to less dependency on these small input structure differences.

A more detailed description of each test case, including what is known from experimental and structural studies about the factors that influence binding differences in the test cases, as well as the success of the methods in reproducing these factors, is provided in the Information S1.

### Conclusion

We developed a pipeline of molecular modeling tools named PocketOptimizer. The program can be used to predict affinity altering mutations in existing protein binding pockets. For enzyme design applications it can be combined with a program such as ScaffoldSelection [24]. In PocketOptimizer receptor-ligand scoring functions are used to assess binding. For its evaluation, we compiled a benchmark set of proteins for which crystal structures and experimental affinity data are available and that can be used to test our and other methodologies. We subjected PocketOptimizer as well as the state-of-the-art method Rosetta to our benchmark test. The overall performance of both approaches was similar, but in detail both had different benefits. Rosetta handles the conformational modeling of the binding pocket better, while PocketOptimizer has the advantage in predicting which of a pair of mutants of the same protein binds the ligand better. This prediction was correct in 66 or 69% of the tested cases using PocketOptimizer (CADDSuite or Vina score, respectively) and in 64% of the cases using Rosetta.

The results show that PocketOptimizer is a well performing tool for the design of protein-ligand interactions. It is especially suited for the introduction of a hydrogen bond if there is an unsatisfied hydrogen donor or acceptor group in the ligand, and for filling voids between the protein and the ligand to improve vdW interactions. For affinity design problems that require a more complex rearrangement of the binding pocket, e.g. a mutation making room for another side chain to interact with the ligand, none of the tested methods appear to perform well.

There are also some other obvious effects that can influence binding, but that are not addressable with the current methods, e.g. protein dynamics or rearrangements of the backbone. Such problems are probably harder to address than the more complicated test cases dealt with in this study, so that we do not expect that current methods can tackle them with much success. Some apparent problems of PocketOptimizer, however, such as the occurrence of unresolvable steric clashes between ligand and side chains should be mendable by better sampling of the conformational space and the introduction of backbone flexibility [36] [37]–[38]. It is conceivable that a continuous minimization step at the end of the design calculation could also be beneficial.

In conclusion, it seems that although PocketOptimizer performs well, and even better in some respects than the state-of-the-art method Rosetta, there is still room for improvement in computational design of protein-ligand binding. Our study highlights the usefulness of benchmark data sets and systematic testing in order to arrive at an informed assessment of computational design methods. In fact it would be interesting to test other available protein design schemes using our benchmark. A comparison of their performance should be very informative. Further, the benchmark will be useful in future test of parts of our modular design pipeline, e.g. by exchanging the force-field in PocketOptimizer its contribution can be tested rather than the overall design approach.

When we started to compile our benchmark set, we were hoping for considerably more test cases. The fact that out of the 6,005 protein structures currently contained in the PDBbind database, only ten suitable test cases could be extracted (twelve if the double cases of neuroaminidase and streptavidin are counted), was rather surprising to us. This emphasizes the need for more benchmark data. Thus, an explicit effort to systematically create experimental and structural data is required. For protein-ligand interaction design it would be desirable to have data that covers many mutations of several pocket positions, ideally also of a set of different proteins.

## Materials and Methods

### Benchmark Set

The basis for the benchmark set is the PDBbind database. It contains a set of crystal structures of proteins complexed with small ligands, and the corresponding experimentally determined binding affinity. [34]. Our analysis is based on release 2010. First, we aligned the sequences of all proteins in the database to each other, using the Needleman-Wunsch algorithm [39] as implemented in the EMBOSS suite [40]. The proteins were then clustered with single linkage clustering, a link was assumed if the sequence identity was ≥95%. One cluster was assumed to contain structures of variants of the same protein with some mutations. Several descriptors were calculated for the protein-ligand complexes. If the crystal structure contains water molecules in the binding pocket, waters that have a high probability to play a role in binding were identified and counted. This was done with the tool WaterFinder included in CADDSuite [28]–[41] that estimates the strength of binding of a water molecule observed in a crystal structure to the protein. The number of rotatable bonds in the ligand is used as a measure of ligand size and flexibility. The ligands of all proteins in a cluster were pairwise compared using ligand fingerprints as implemented in OpenBabel [42] to measure their similarity and identity. For protein pairs of the same cluster with identical ligands, the pockets as defined by PDBbind were investigated for any mismatches corresponding to mutations. To identify suitable protein pairs, we searched our dataset for protein variants within a cluster that (1) have the same ligand bound, (2) contain at least one mutation in the binding pocket, (3) have no mutations elsewhere, (4) contain less than four water molecules potentially involved in binding, and (5) have a ligand with less than 15 rotatable bonds. As the results contained mostly single mutants, an additional search was performed looking for mutants with (1) at least two mutations in the pocket, (2) no mutations elsewhere, (3) allowing for less than 15 rotatable ligand bonds and (4) less than 7 potential binding waters molecules. The proteins identified by these searches were investigated further by visually inspecting their structure and looking at the corresponding literature. Suitable proteins were included in our set. Reasons for rejecting a protein were large conformational differences of the backbone in the binding pocket, the fact that affinity differences between variants is not caused by any protein-ligand interaction, but for example by changes in protein dynamics, and missing atoms of residues in the binding pocket in a crystal structure.

### Design Pipeline PocketOptimizer

A diagram of the PocketOptimizer workflow is shown in Figure 1. The backbone conformation of the protein stays fixed in the calculations, as do the side chain conformations of residues that do not contact the ligand or a residue that is mutated between variants. Amino acid side chain flexibility is sampled by a conformer library we compiled for this purpose [25]–[27]. For this, a set of high-quality protein structures from the PDB was selected by requiring a maximal resolution of 1.2 Å at least 40 residues, no CAVEAT record. Hydrogen atoms were added using reduce [43]. Side chain conformers of these structures were further filtered by requiring a temperature factor below 30, no alternative conformations and no overlaps with other atoms in the structure according to probe [44]. The conformers were superimposed at the backbone atoms and clustered as described in reference [22], resulting in 2211 conformers. The generation of ligand conformers and binding pocket poses also closely follows reference [22]. Ligand conformers are created with omega2 by OpenEye Software [45]. These are superimposed onto the ligand in the crystal structure, rotated around 6 approximately equally distributed axes through the ligand center of mass, and translated in x, y, z directions. The resulting ligand poses are filtered to exclude poses with obvious clashes with the protein backbone.

Binding energy scores between protein and ligand are calculated by a receptor-ligand scoring function. The first one is contained in CADDSuite [28]. It is composed of terms for electrostatic, vdW, solvation and hydrogen bond energy scores. The second score used by PocketOptimizer is Autodock Vina [29]. Protein packing energies are calculated using the AMBER force field [31] with electrostatics scaled by a factor of 0.01. In order to be compatible with the energy score optimization algorithm, the energy values have to be pairwise decomposable, i.e. of the form 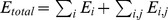. 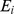 are the self energies of the variables (side chain conformers or ligand poses), i.e. their inherent energies and the energies with the fixed protein parts, and 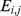 the pairwise energies between the variables. As we are interested in improving binding affinity, we chose to upscale the binding energies by a factor of ten for CADDSuite scores and a factor of 100 for Autodock Vina scores to arrive at absolute values that are in the same range as the AMBER packing energies. The 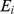 and 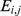 energy tables are computed for all side chain conformers at the pocket positions and the ligand poses. The problem of finding the minimum energy conformation is formulated in graph-theroretic terms [32] and solved using the MPLP algorithm by Sontag et al. [33]. The energy minimum identifies the best design with corresponding score values and conformation.

PocketOptimizer is realized as a collection of binaries and scripts that perform the different subtasks. It was developed and tested on Ubuntu Linux 10.04 operating system. AMBER packing energy calculations are implemented in C++ using BALL [41], so is the ligand pose generation tool. Protein-ligand energies for CADDSuite are calculated with a scorer binary implemented in C++ as well, vina energies are calculated using the vina binary provided with the Autodock vina software distribution. The side chain conformer library contains the structures of the amino acid side chains in PDB and SDF formats. Several Python scripts are provided that interface between the different parts and allow a convenient conducting of a protein design task with the PocketOptimizer pipeline. Intermediate result are stored in standard file formats, SDF and PDB formats for structural data, and CSV files for energy tables. This allows the user to easily inspect this data with standard tools. It also facilitates the possibility to use a different approach for one of the modules, e.g. a different docking function, while the rest of the pipeline can remain unaltered.

### Setup for PocketOptimizer Benchmark

The protein structures were briefly minimized using Chimera’s [46] AMBER implementation. Amino acids of the binding pocket positions that were allowed to change conformations in the calculations had to have a distance smaller than 4 Å of at least one side chain atom to the ligand or to one of the residues that are mutable. Ligand conformers were rotated by ±20° around each axis and translated by 0.5 Å in each direction to create the ligand poses. If this resulted in more than 3000 poses, the conformers were filtered by similarity to the crystal structure conformation until meeting the max 3000 poses criterion. For proteins that contain metals in their binding pocket that are coordinated by the ligand, the ligand poses were filtered for poses that are geometrically compatible for coordination.

### Rosetta Design Setup

The Rosetta enzyme design application as implemented in Rosetta 3.3 [30] was used with parameters closely following the relevant documentation. Protein structures were briefly minimized using the Rosetta receptor preparation application provided for this task, generating ten resulting structures of which the one with the best energy was used for the design runs. Ligand conformers were generated using omega2, ligand charges added with the quacpac program of OpenEye software [45], and Rosetta ligand params files generated with the provided molfile_to_params python script as included in the 3.3 distribution. No catalytic constraints were used for the enzyme design application runs, effectively making it a receptor design application. 1000 designs were created for every protein and every mutation on that protein with experimental affinity data in the test set. The best design was determined by the ranking scheme suggested in the documentation, it is the design with the best predicted binding energy among the designs with the 10% top total scores.

## Supporting Information

Information S1 (PDF)
