# Supplementary material for: Binding Pocket Optimization by Computational Protein Design
Source: PLoS One. 2012 Dec 27;7(12):e52505. doi: 10.1371/journal.pone.0052505 (PMC3531388; doi:10.1371/journal.pone.0052505)
Supplement: Information S1 — (PDF) [file pone.0052505.s001.pdf]

Detailed analysis of protein-ligand binding benchmark

In the following, we discuss each benchmark protein. A summary of what is known from experimental and structural studies about the factors that influence binding differences in the different variants is provided. This is then used to investigate if the design runs could reproduce these factors. The analysis is based on the top-ranked design for each run: For PocketOptimizer, this is the design with the best total score; for Rosetta, it is the design with the best binding score among the 10% best total score designs.

Carbonic Anhydrase II

For carbonic anhydrase II it has been suggested that ligand affinity is modulated by side chain charge and hydrophobicity at sequence position 198. Hydrophobic amino acids Phe, Trp and Leu yield more or less the same affinity and are favored, while negatively charged Glu has unfavorable electrostatic effects with the negatively charged ligand [1].

The designs of PocketOptimizer show a distinct dependence of the predicted energies on the crystal structure used as scaffold, more so than in other test cases. Design energy scores for scaffold 1ydb, which is the crystal structure of variant 198F, follow the affinity logarithm more closely (see Figure 4). A steric clash (i.e. a high van-der-Waals (vdW) energy value) between the Phe side chain and the ligand in designs based on 1yda and 1ydd is predicted for both receptor-ligand scoring functions. A small clash with the Trp side chain for 1yda 198W designs, and a small clash of a neighboring side chain presumably displaced by the large Trp residue 1ydd 198W designs can also be identified. We suspect that insufficient sampling of the conformational space, especially of the ligand position, lead to this situation where no conformation can be found that omits vdW clashes. While the value of the electrostatic component of the CADDSuite energy score for 198E designs is higher than for the other variants, the overall contribution of this component to the score is so small that it does not influence the outcome.

Rosetta does not exhibit this dependence on the scaffold structure, and no obious vdW clashes between Phe 198 and ligand are present. However, there is a higher vdW repulsion energy in designs based on 1yda and 1ydd than in 1ydb, suggesting that there is some steric strain in these structures. The Phe 198 side chains in 1ydd and 1yda designs are further away from the ligand as in the 198F crystal structure, so the native interactions could not be reproduced. The RMSD values between designs and crystal structure ligand poses are as high for Rosetta as they are for PocketOptimizer, suggesting that for this test case the ligand modeling is quite hard.

D7r4 amine-binding protein

This test case (as well as the estrogen receptor case) was derived from an earlier literature search before we started searching the PDBbind database systematically. For this reason, only one protein-ligand complex crystal structure exists. The amine binding case is also the only one where one variant, namely 111L, does not bind its ligand in a detectable range. 111L loses a crucial hydrogen bond to its ligand tryptamine, which is usually formed by the Asp in 111D, thus disrupting a hydrogen bond network that stabilizes the position of the amino group of tryptamine [2].

The RMSD of the design ligand pose to the crystal structure has a value of 0 Å (see Figure 3), which is possible because our ligand modeling approach always includes the position of the ligand in the scaffold structure. For all PocketOptimizer designs of variant 111D, this native position was recovered. The hydrogen bond between Asp 111 and the ligand is also recovered in all designs. In comparison Rosetta succeeds as well in reproducing this crucial hydrogen bond.

Estrogen receptor α

In this test case, which is the second derived from a literature search, there is only one crystal structure available, namely the one of the best binding variant 353E. Both the 353E and 353Q variants form hydrogen bonds to the ligand estradiol, the one formed by 353Q is weaker than by 353E. Arg 394 also forms an hydrogen bond to the same ligand hydroxyl group [3].
These hydrogen bonds are both found in PocketOptimizer designs. Vina predicts the Gln hydrogen bond to be slightly stronger than the one formed by Glu, while the CADDSuite correctly prefers the Glu hydrogen bond. Rosetta succeeds as well in reproducing the hydrogen bond between 353E and the ligand. However, it does not predict an hydrogen bond between Gln in variant 353Q, the carboxyl group of the Gln side chain is pointing away from the ligand. None of the Rosetta designs find the correct conformer for Arg 394, and consequently do not predict its hydrogen bond with the ligand.

HIV-1 protease

DMP323 is the largest ligand in our test set, and the HIV-1 protease pocket is the largest by residue count. The pocket is formed symmetrically by both chains of the HIV-1 homodimer, consequently the four mutable positions are two positions that are mirrored between the two chains. In both variants with Val at position 84, the missing $C_{\delta 1}$ atom of Ile at this position diminishes vdW contacts to the ligand, as the $C_{\delta 1}$ "hole" is not filled by conformational rearrangements. The two variants with Phe at position 82 behave differently: in the FIFI (82F, 84I) variant, Phe forms additional vdW interactions with the ligand. In variant FVFV (82F, 84V), the Phe side chain is rotated compared to in FIFI, and does not contact the ligand extensively. The backbone at position 82 in both variants with Phe moves 0.5 Å compared to 82V variants to accommodate the larger side chain [4].
The differences in binding scores predicted by the PocketOptimizer- CADDSuite score designs are mostly created by different vdW and solvation scores. The loss of favorable vdW contacts when mutating Ile 84 to Val is reproduced correctly. Variant VVVV is judged to be the worst binder by both scoring functions on all scaffolds because at position 82 Val is predicted to contact the ligand less and shield it less from solvent than Phe. The two different conformations of Phe for variants FVFV and FIFI are not found in the CADDSuite score designs, both are predicted to have beneficial vdW and solvation interactions. Binding score differences in designs using Vina are also mainly based on hydrophobic interactions. The benefit of the larger Phe side chain is not as overestimated as with CADDSuite score, leading to a better reconstruction of the affinity differences (see Figure 4). The shape of the binding pocket is rather complex with several subpockets. This is probably the reason why variants designed on their native crystal structure all chose the native ligand conformation.
Rosetta on the other hand predicts variant VIVI to be the best binder on all scaffold structures, and also to have the best total energy score. FVFV is correctly determined to be the worst binder. FVFV and FIFI have the worst total scores. The Phe 82 side chain conformers in the designs of variants FIFI and FVFV are not reproduced well, especially for FIFI designs. They tend to point away from the ligand and towards the solvent. Rosetta behaves inversly to PocketOptimizer using the CADDSuite score: it underestimates the benefit of Phe 82 for binding in FIFI designs, while correctly predicting that Phe 82 is not involved in binding in FVFV. The CADDSuite score correctly predicts the binding contacts in FIFI, while failing to predict the lack of contact in FVFV.

Ketosteroid isomerase

In both ketosteroid isomerase variants, Asp and Asn residues at position 40 have equivalent conformations. Both interact with the ligand via hydrophobic interactions, the negative charge of Asp diminishes binding [5].
PocketOptimizer is able to reproduce the order of affinity in all designs. Asn 40 is predicted to have

slightly better vdW interactions with the ligand, and the CADDSuite score electrostatics component value is higher for Asp than for Asn. The side chain conformations are also reproduced well (Figure 3). Rosetta also succeeds in reproducing the correct side chain conformations. The correct order of the mutants based on binding energy score is only predicted for scaffold structure 1oh0 of the 40D variant, however, the binding scores are virtually the same for both variants 40D and 40N. The difference in binding energy between 1ogx 40D and 40N designs are mainly due to the fact that the ligand in 40D fails to form a hydrogen bond in a pocket region away from position 40, which the ligand achieves in 40N.

Lectin II

In Lectin II the better binding affinity of variant AG is caused by the Ala of variant 22A, which allows the ligand to form a hydrogen bond to Asp 96. A hydroxyl group of the ligand is rotated with respect to variant SN to realize this hydrogen bond. In variant SN, the side chain of Ser 22 is in the way and blocks this interaction. 24N could form a weak hydrogen bond to the ligand, but it is not entirely clear if it does [6].
In this case PocketOptimizer fails to reproduce the pocket rearrangement, and consequently ranks the variants incorrectly by the predicted binding score. Both CADDSuite and Vina scores predict hydrogen bonds to Asn 24 in the SN designs (Vina also to Ser 22 for one scaffold), as well as better vdW interaction and solvation energies between the bulkier side chains Ser and Asn when compared to Ala and Gly.
In comparison Rosetta predicts the correct order for the binding scores only for designs on scaffold structure 2jdy. It also fails to predict the conformational changes of the ligand upon mutation. In all designs, the ligand has the same conformation as in the scaffold crystal structure the design is based upon, independent of the introduced mutations.

Methylglyoxal synthase

In methylglyoxal synthase all three residues at position 98 form an hydrogen bond to the ligand. The pocket conformations and the ligand positions are very similar in all crystal structures. The reason for the loss of affinity in the 98Q mutant is not really clear [7].
PocketOptimizer fails to recover the correct order by binding affinity of the three mutants. Only in the native scaffold 98N, 1s89, a strong hydrogen bond is predicted between the Asn and the ligand, consequently on this scaffold 98N is predicted to be the best binder. On the other scaffolds, the hydrogen bond is missed or in one case predicted to be very weak, thus predicting 98N to be the worst binder. Both CADDSuite and Vina scores predict hydrogen bonds of 98Q to the ligand in all designs, which is therefore predicted to bind best in scaffolds 1s89 and 1egh.
In this case Rosetta also fails to predict the correct order based on the binding score. As with PocketOptimizer, the predictions for designs on scaffold 1s89 are different to the other ones, although the scaffold structures are very similar (backbone RMSDs of 0.2 Å). Variant 98N is deemed the best binder, whereas for the designs based on the other two scaffolds 98N is the worst binder. This is also caused by the failure to predict the hydrogen bond from 98N to the ligand in 1s8a and 1egh-based designs.

Neuroaminidase

There are two single mutants of neuroaminidase that are used as separate test cases. The first is position 274, which is mutated from His to Tyr. Position 274 is relatively far away from the ligand, the closest two atoms of His and ligand in the 274H variant are 6 Å apart. Consequently, there is no significant direct interaction between them. The diminished binding in the 274T variant is caused by the rearrangement of the carboxy group of Glu 276, which is caused by the bulkier Tyr 274 side chain. The charged carboxyl group of Glu 276 is pushed to a formerly hydrophobic binding pocket, where it is detrimental

to binding [8].
PocketOptimizer was unable to find a Tyr conformer for position 274 that did not lead to large vdW clashes in the protein pocket for scaffold 2hu4 (the crystal structure of the 274H variant). Consequently, no sensible designs could be calculated. The designs on scaffold 3cl0 are not ordered correctly by the predicted binding scores, and the rearrangement of Glu 276 is not reproduced.
Rosetta also fails to predict the correct binding order. The rearrangement of Glu 276 is not correctly reproduced. It keeps its crystal structure conformation for designs on scaffold 3cl0, while it assumes a conformation in 2hu4 designs not seen in any of the crystal structures.

The second test case position is 294, which has variants Asn and Ser. The change in binding affinity is also due to rearrangements in the binding pocket. Asn and Ser at position 294 point in opposite directions, Asn interacts with Tyr 347, the loss of this interaction in the 294S variant weakens the hydrogen bond of Tyr 347 to the ligand. Additionally, the hydroxyl group of Ser 294 is located in a site that is hydrophobic in the 294N variant, further weakening the interaction to the ligand [8].
Both scoring variants used within PocketOptimizer fail to reproduce these conformational rearrangements. Rosetta correctly orders the designs on both scaffolds based on predicted binding energies. However, the Tyr 347 interaction with 294N is not reproduced, Tyr 347 points in a different direction in the designs than in the crystal structures. In designs on scaffold 3cl2, the predicted energies for 194N and 194S are very similar, so the affinity improvement of 249N is not predicted convincingly.

Purine nucleoside phosphorylase

In this case the mutable position 257 is at the entry of the binding pocket with contact to the solvent. Variants 257H and 257D form hydrogen bonds to the ligand. The side chain of 257F points away from the ligand, it does not contact it directly, neither does 257G. It is not clear what leads to the affinity differences. The position on the binding pocket entry together with the relatively high temperature factors at this position in all variants suggests that there is some influence on the dynamics of binding, perhaps facilitating or hindering the entry of the ligand into the pocket [9].
PocketOptimizer correctly predicts hydrogen bonds of 257H and 257D to the ligand, with the exception of the design of 257D using the CADDSuite score on scaffold 1rsz, which explains the relatively bad binding score (cmp. Figure 4). 257G is always predicted to be the worst binder, lacking the beneficial direct vdW interactions with the ligand. The orientation of the side chain of 257F is different to the one in the crystal structure, it is rotated towards the ligand and therefore adds vdW and solvation energy contributions.
Rosetta's ordering of the variants by binding energy is different for each scaffold structure used. Only for 2a0w it resembles the order by affinity. The hydrogen bonds of the ligand to 257H is not found in any of the designs. Only in one design, on scaffold 2a0y, the Asp is in a conformation that could form an hydrogen bond with the ligand, however it seems not to be recognized as an energetically favorable interaction. The predicted binding energies are very similar for all variants among the designs on each scaffold.

Streptavidin

There are mutants at two positions of streptavidin, which lead to two test cases. Asn at position 23 in variant 23N forms a hydrogen bond to the ligand, which is lost in 23A; the smaller Ala side chain also leaves a void [10].
In this case PocketOptimizer succeeds in reproducing the 23N hydrogen bond to the ligand. It also predicts a somewhat weaker hydrogen bond between 23E and the ligand for scaffold 1n43 with both scoring functions. There is no structural or experimental data that can confirm the existence of this interaction.

The Asn hydrogen bonds are also correctly predicted by Rosetta, while an hydrogen bond between Glu in variant 23E and the ligand is not found. The ranks of the variants by binding score are different for the two scaffold structures. The predicted side chain conformations for Glu 23 are also different on the two scaffolds. For 1swe, the best predicted binder is 23N, with 23E as the worst. For 1n43, 23E has the best binding score, while 23A is the worst.

The second variable position 27 also forms a hydrogen bond to the ligand in its variant 27S, which is lost in 27A [10].
This hydrogen bond is correctly predicted for PocketOptimizer designs.
Rosetta predicts in the 27S design on scaffold 1swe a hydrogen bond of the Ser to the ligand. 27S is also correctly predicted as the better binder. On scaffold 1n9m, the Ser 27 side chain has a different orientation, and does not form an hydrogen bond with the ligand. Here, 27S is incorrectly predicted to be a worse binder than 27A.

Thymidylate synthase

In thymidylate synthase variant 229D forms a hydrogen bond to the ligand. Atom $N_{\delta 2}$ of Asn 229 in variant 229N also would have the right distance and geometry for a hydrogen bond, but it is unlikely that it forms, because both $N_{\delta 2}$ and the putative binding partner atom in the ligand are hydrogen donors. Asn 229 is also sterically unfavorable. The smaller side chain of Cys at position 229 does not have these steric problems [11].
In this case PocketOptimizer fails to predict the correct order of these variants by affinity. It predicts hydrogen bonds of both 229D and 229N to the ligand in most cases, these two side chains also benefit from better vdW and solvation score contributions. The bad performance of Cys is therefore due to less predicted contact with the ligand, leading to smaller vdW and solvation contributions.
The order of variants by predicted binding energy for Rosetta designs is different for all three scaffold structures. For scaffolds 1njc and 1nje, variant 229D is the best binder because the hydrogen bond to the ligand is correctly predicted. For scaffold 1nja, Rosetta misses the hydrogen bond and consequently ranks variant 229D to be the worst binder. A hydrogen bond between Asn 229 in variant 229N is predicted for designs on scaffolds 1njc and 1nja, for the latter it is therefore predicted as the best binder. For scaffold 1nje, 229N is predicted to be the worst binder.

Trypsin

The experiment that provided the data for this test case sought to answer whether the carboxy group that interacts strongly with the ligand in wild type variant 189D/226G could be provided in the same or similar spacial position in the binding pocket by “attaching” it to a different backbone position. This is why spatially close residues 189 and 226 were switched. In the wild type variant 189D/226G, Asp 189 forms two hydrogen bonds, one to each of the benzamidine nitrogens. Switched variant 189G/226D has a lower affinity to the ligand, 226D only forms one hydrogen bond to the ligand, which is rotated compared to its pose in variant 189D/226G [12].
PocketOptimizer predicts the correct binding score order only for scaffold 1ane of 189D/226G. Designs for 189D/226G correctly predict hydrogen bonds from Asp 198 to the ligand. The designs of 189G/226D in this scaffold predict vdW clashes of the ligand with some pocket side chains. It fails to find the correct ligand pose in 189D/226G designs on scaffold 1bra of 189G/226D, and consequently does not predict any hydrogen bond of Asp 189 to the ligand.
In Rosetta designs, the correct order by binding score is also only predicted for scaffold structure 1ane. The repositioning of the ligand could not be predicted in the two designs using the crystal structure of the other variant as a scaffold. The strong hydrogen bonds between 198D and the ligand are not found in the corresponding design on 1bra.

Table S1. Scaled energy score values of benchmark designs.

**Design Method**	**Scaffold**	**Mutations**				
		**Carbonic anhydrase II**				
		F	W	L	R	E
PocketOptimizer (CADDSuite)	1ydb	0.564	0.0	1.0	0.755	0.871
	1ydd	1.0	0.0	0.502	0.226	0.478
	1yda	0.621	1.0	0.019	0.0	0.0
PocketOptimizer (Vina)	1ydb	0.432	0.0	1.0	0.576	0.834
	1ydd	1.0	0.0	0.309	0.105	0.25
	1yda	1.0	0.568	0.02	0.036	0.0
Rosetta	1ydb	0.0	0.0	0.5	1.0	0.5
	1ydd	0.0	1.0	1.0	1.0	1.0
	1yda	0.0	0.634	0.317	1.0	0.463
		**D7r4 amine binding protein**				
		D	L			
PocketOptimizer (CADDSuite)	2pql	0.0	1.0			
PocketOptimizer (Vina)	2pql	0.0	1.0			
Rosetta	2pql	0.0	1.0			
		**Estrogen receptor**				
		E	Q	A		
PocketOptimizer (CADDSuite)	1gwr	0.0	0.278	1.0		
PocketOptimizer (Vina)	1gwr	0.101	0.0	1.0		
Rosetta	1gwr	0.0	0.908	1.0		
		**HIV Protease**				
		FIFI	VIVI	VVVV	FVFV	
PocketOptimizer (CADDSuite)	1met	0.0	0.529	1.0	0.42	
	1mes	0.0	0.757	1.0	0.254	
	1meu	0.0	0.492	1.0	0.327	
PocketOptimizer (Vina)	1met	0.0	0.164	1.0	0.836	
	1mes	0.134	0.0	0.866	1.0	
	1meu	0.0	0.392	1.0	0.586	
Rosetta	1met	0.396	0.0	0.547	1.0	
	1mes	0.318	0.0	0.379	1.0	
	1meu	0.779	0.0	0.384	1.0	
		**Ketosteroid isomerase**				
		N	D			
PocketOptimizer (CADDSuite)	1ogx	0.0	1.0			
	1oh0	0.0	1.0			
PocketOptimizer (Vina)	1ogx	0.0	1.0			
	1oh0	0.0	1.0			
Rosetta	1ogx	1.0	0.0			
	1oh0	0.0	1.0			

Table S1 – continued on next page

Table S1 – continued from previous page

Design Method	Scaffold	Mutations		
		**Lectin II**		
		AG	SN	
PocketOptimizer (CADDSuite)	2jdn	1.0	0.0	
	2jdy	1.0	0.0	
PocketOptimizer (Vina)	2jdn	1.0	0.0	
	2jdy	1.0	0.0	
Rosetta	2jdn	1.0	0.0	
	2jdy	0.0	1.0	
		**Methylglyoxal synthase**		
		H	N	Q
PocketOptimizer (CADDSuite)	1egh	0.849	1.0	0.0
	1s89	1.0	0.0	0.382
	1s8a	0.393	1.0	0.0
PocketOptimizer (Vina)	1egh	0.652	1.0	0.0
	1s89	1.0	0.0	0.054
	1s8a	0.125	1.0	0.0
Rosetta	1egh	0.351	1.0	0.0
	1s89	1.0	0.0	0.604
	1s8a	0.0	1.0	0.076
		**Neuroaminidase 1**		
		H	Y	
PocketOptimizer (CADDSuite)	2hu4	–	–	
	3cl0	–	–	
PocketOptimizer (Vina)	2hu4	–	–	
	3cl0	–	–	
Rosetta	2hu4	1.0	0.0	
	3cl0	1.0	0.0	
		**Neuroaminidase 2**		
		N	S	
PocketOptimizer (CADDSuite)	2hu4	0.0	1.0	
	3cl2	1.0	0.0	
PocketOptimizer (Vina)	2hu4	1.0	0.0	
	3cl2	1.0	0.0	
Rosetta	2hu4	0.0	1.0	
	3cl2	0.0	1.0	

Table S1 – continued on next page

Table S1 – continued from previous page

Design Method	Scaffold	Mutations			
**Purine nucleoside phosphorylase**					
		H	G	D	F
PocketOptimizer (CADDSuite)	1rsz	0.0	1.0	0.542	0.238
	2a0w	0.305	1.0	0.0	0.215
	2a0y	0.0	1.0	0.042	0.221
	2a0x	0.0	1.0	0.083	0.315
PocketOptimizer (Vina)	1rsz	0.111	1.0	0.359	0.0
	2a0w	0.0	1.0	0.083	0.239
	2a0y	0.14	1.0	0.196	0.0
	2a0x	0.0	1.0	0.331	0.089
Rosetta	1rsz	1.0	0.833	0.0	0.833
	2a0w	0.0	0.5	0.5	1.0
	2a0y	0.0	1.0	0.0	0.0
	2a0x	1.0	1.0	0.5	0.0
**Streptavidin 1**					
		N	E	A	
PocketOptimizer (CADDSuite)	1swe	0.0	0.572	1.0	
	1n43	0.0	1.0	0.622	
PocketOptimizer (Vina)	1swe	0.0	0.936	1.0	
	1n43	0.0	1.0	0.738	
Rosetta	1swe	0.0	1.0	0.412	
	1n43	0.25	0.0	1.0	
**Streptavidin 2**					
		S	A		
PocketOptimizer (CADDSuite)	1swe	0.0	1.0		
	1n9m	0.0	1.0		
PocketOptimizer (Vina)	1swe	0.0	1.0		
	1n9m	0.0	1.0		
Rosetta	1swe	0.0	1.0		
	1n9m	1.0	0.0		
**Thymidilate synthase**					
		C	D	N	
PocketOptimizer (CADDSuite)	1nja	1.0	0.376	0.0	
	1njc	1.0	0.477	0.0	
	1nje	1.0	0.446	0.0	
PocketOptimizer (Vina)	1nja	1.0	0.411	0.0	
	1njc	1.0	0.26	0.0	
	1nje	1.0	0.058	0.0	
Rosetta	1nja	0.976	1.0	0.0	
	1njc	1.0	0.0	0.069	
	1njc	0.44	0.0	1.0	

Table S1 – continued on next page

Table S1 – continued from previous page

**Design Method**	**Scaffold**	**Mutations**	
	**Trypsin**		
		DG	GD
PocketOptimizer (CADDSuite)	1ane	0.0	1.0
	1bra	1.0	0.0
PocketOptimizer (Vina)	1ane	0.0	1.0
	1bra	1.0	0.0
Rosetta	1ane	0.0	1.0
	1bra	1.0	0.0

This table lists all predicted binding score values of the top designs calculated for the benchmark evaluation. Each section of the table contains the data for one test case. Energy scores are scaled to the range 0 to 1 for easy comparison between methods. The lowest predicted binding score of designs calculated with one method and on one scaffold crystal structure set to 0, the highest to 1. The PDB IDs of the scaffold crystal structures are given in column Scaffold. Compare Table 1 for details on the scaffolds and for the experimental affinities of the mutational variants. Figure S1 plots these values for all test cases with more than three mutational variants and allows visual inspection and comparison of the designs.

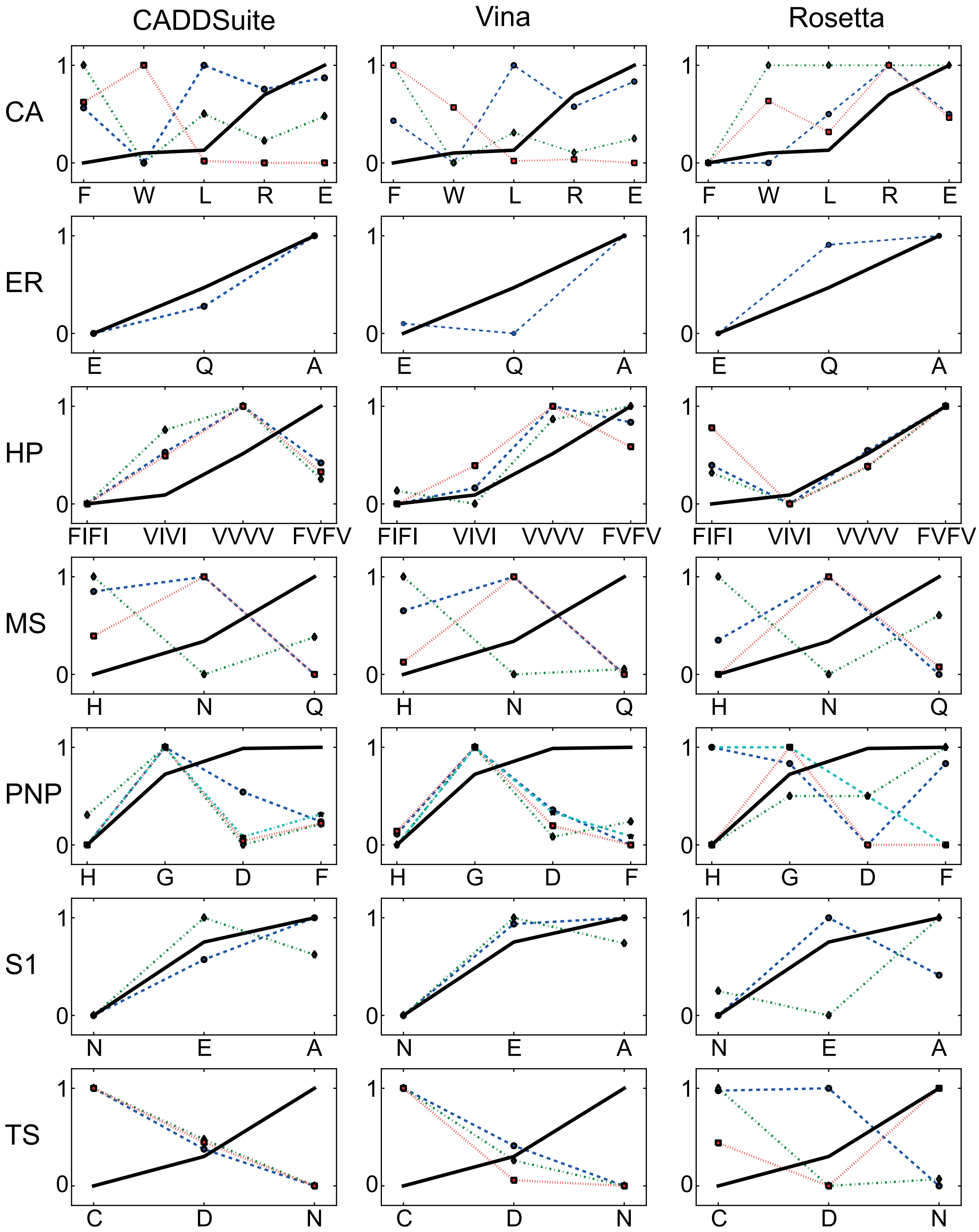

Figure S1. Comparison of the energy scores versus the affinities of the mutations show how well the programs reproduce the differences. (Continued on next page.)

Figure S1. (continued) For each test case with more than two mutations, this figure contains plots of the top binding scores of CADDSuite, Vina, and ROSETTA designs for each mutation on each scaffold structure together with the logarithm of the affinity. Values are scaled to fit in the same range. Shown on the x-axis of a plot are the mutants in order of affinity to the ligand (the leftmost has the lowest affinity, compare Table 1 for the actual values). The y-axis measures predicted binding scores for the designs, and the log affinities, scaled between 0 and 1. Both are proportional to the binding free energy, and can therefore be compared when scaled to the same range. The lowest predicted binding score or log affinity is set to 0, the highest respective value to 1. Each plot contains a line for the affinity logarithm (solid, black no marker). This line represents the goal, if a method predicts binding well, the binding score lines should closely follow the log affinity line. The other markers and lines show the scaled predicted binding scores. One line represents the designs calculated for all available mutants, calculated by using one crystal structure as the scaffold. (Crystal structure 1: dashed, blue, circle markers; structure 2: red, dotted, square markers; structure 3: green, dash-dot pattern, diamond markers; structure 4: cyan, two dashes one dot pattern, star markers). We chose to use lines for representation, because this makes it easy to visually compare the shape of the black log affinity line to the lines representing the design binding scores. Each row has plots for one test case, in parentheses the order of scaffold structures is listed: *CA*: Carbonic anhydrase II (1ydb, 1yda, 1ydd). *ER*: Estrogen receptor α (1gwr); *HP*: HIV-1 protease (1met, 1meu, 1mes), *MS*: Methylglyoxal synthase (1egh, 1s8a, 1s89), *PNP*: Purine nucleoside phosphorylase (1rsz, 2a0y, 2a0w, 2a0x), *S1*: Streptavidin test 1 (1swe, 1n43), *TS*: Thymidilate synthase (1nja, 1nje, 1njc).

References

1. Nair SK, Krebs JF, Christianson DW, Fierke CA (1995) Structural basis of inhibitor affinity to variants of human carbonic anhydrase ii. Biochemistry 34: 3981–3989.

2. Mans BJ, Calvo E, Ribeiro JMC, Andersen JF (2007) The crystal structure of d7r4, a salivary biogenic amine-binding protein from the malaria mosquito anopheles gambiae. J Biol Chem 282: 36626–36633.

3. Chen Z, Katzenellenbogen BS, Katzenellenbogen JA, Zhao H (2004) Directed evolution of human estrogen receptor variants with significantly enhanced androgen specificity and affinity. J Biol Chem 279: 33855–33864.

4. Ala PJ, Huston EE, Klabe RM, McCabe DD, Duke JL, et al. (1997) Molecular basis of hiv-1 protease drug resistance: structural analysis of mutant proteases complexed with cyclic urea inhibitors. Biochemistry 36: 1573–1580.

5. Kim SW, Cha SS, Cho HS, Kim JS, Ha NC, et al. (1997) High-resolution crystal structures of delta5-3-ketosteroid isomerase with and without a reaction intermediate analogue. Biochemistry 36: 14030–14036.

6. Adam J, Pokorn M, Sabin C, Mitchell EP, Imberty A, et al. (2007) Engineering of pa-iil lectin from pseudomonas aeruginosa - unravelling the role of the specificity loop for sugar preference. BMC Struct Biol 7: 36.

7. Marks GT, Susler M, Harrison DHT (2004) Mutagenic studies on histidine 98 of methylglyoxal synthase: effects on mechanism and conformational change. Biochemistry 43: 3802–3813.

8. Collins PJ, Haire LF, Lin YP, Liu J, Russell RJ, et al. (2008) Crystal structures of oseltamivir-resistant influenza virus neuraminidase mutants. Nature 453: 1258–1261.

9. Murkin AS, Birck MR, Rinaldo-Matthis A, Shi W, Taylor EA, et al. (2007) Neighboring group participation in the transition state of human purine nucleoside phosphorylase. Biochemistry 46: 5038–5049.

10. Trong IL, Freitag S, Klumb LA, Chu V, Stayton PS, et al. (2003) Structural studies of hydrogen bonds in the high-affinity streptavidin-biotin complex: mutations of amino acids interacting with the ureido oxygen of biotin. Acta Crystallogr D Biol Crystallogr 59: 1567–1573.

11. Finer-Moore JS, Liu L, Schafmeister CE, Birdsall DL, Mau T, et al. (1996) Partitioning roles of side chains in affinity, orientation, and catalysis with structures for mutant complexes: asparagine-229 in thymidylate synthase. Biochemistry 35: 5125–5136.

12. Perona JJ, Tsu CA, McGrath ME, Craik CS, Fletterick RJ (1993) Relocating a negative charge in the binding pocket of trypsin. J Mol Biol 230: 934–949.
